# Seasonal variation in equine follicular fluid proteome

**DOI:** 10.1186/s12958-019-0473-z

**Published:** 2019-03-06

**Authors:** G. A. Dutra, G. M. Ishak, O. Pechanova, T. Pechan, D. G. Peterson, J. C. F. Jacob, S. T. Willard, P. L. Ryan, E. L. Gastal, J. M. Feugang

**Affiliations:** 1 Department of Animal Science, Food and Nutrition, Southern Illinois University, Carbondale, IL USA; 2 Department of Reproduction and Animal Evaluation, Federal Rural University of Rio de Janeiro, Seropédica, Brazil; 3 Department of Surgery and Obstetrics, College of Veterinary Medicine, University of Baghdad, Baghdad, Iraq; 4 Institute for Genomics, Biocomputing and Bioinformatics, University, Mississippi State, Oxford, MS USA; 5 Department of Animal and Dairy Sciences, Mississippi State University, Mississippi State, 4025 Wise Center, PO Box 9815, Mississippi State, MS 39762 USA

**Keywords:** Ovary, Follicle, Seasonality, Proteomics, Mares, horses

## Abstract

**Background:**

Proteomic studies of follicular fluid (FF) exist for several species, including the horse; however, the seasonal influence on FF proteome has not been explored in livestock. The application of high-throughput proteomics of FF in horse has the potential to identify seasonal variations of proteins involved in follicle and oocyte growth.

**Methods:**

This study (i) profiles the proteomes of equine FF collected from dominant growing follicles during the spring anovulatory season (SAN), and spring (SOV), summer (SUM), and fall (FOV) ovulatory seasons; and (ii) identifies season-dependent regulatory networks and associated key proteins.

**Results:**

Regardless of season, a total of 90 proteins were identified in FF, corresponding to 63, 72, 69, and 78 proteins detected in the SAN, SOV, SUM, and FOV seasons, respectively. Fifty-two proteins were common to all seasons, a total of 13 were unique to either season, and 25 were shared between two seasons or more. Protein-to-protein interaction (PPI) analysis indicated the likely critical roles of plasminogen in the SAN season, the prothrombin/plasminogen combination in SUM, and plasminogen/complement C3 in both SOV and FOV seasons. The apolipoprotein A1 appeared crucial in all seasons. The present findings show that FF proteome of SUM differs from other seasons, with FF having high fluidity (low viscosity).

**Conclusions:**

The balance between the FF contents in prothrombin, plasminogen, and coagulation factor XII proteins favoring FF fluidity may be crucial at the peak of the ovulatory season (SUM) and may explain the reported lower incidence of hemorrhagic anovulatory follicles during the SUM season.

**Electronic supplementary material:**

The online version of this article (10.1186/s12958-019-0473-z) contains supplementary material, which is available to authorized users.

## Background

Within the existing efforts to improve fertility in livestock and companion animals, a different knowledge is sought after to achieve a greater reproduction rate, especially for females. A lack of high-quality oocytes and reliable reproductive biomarkers [1, 2] represents an obstacle toward the success. Meanwhile, the high dynamic in the composition of the follicular fluid (FF) during follicular growth [3, 4] creates an opportunity for the identification of key molecules that are critical for oocyte developmental competence acquisition. Furthermore, the situation is even more challenging in horses, due to the obvious reproductive seasonality [4].

The ovarian activity of non-pregnant mares is continuously changing throughout the year, presenting periods of intense activity during summer, low activity during winter (deep anestrus phase), and irregular activity during spring and fall transitional seasons [5–7]. Results of previous studies have shown differences in preovulatory follicle diameter and blood flow [8–10], and hormonal concentrations among seasons [9]. Studying the effect of season on ovarian activity is important not only for better understanding of the follicular dynamics in mares but also for improving our knowledge regarding the follicular environment and biological processes associated with oocyte maturation and ovulation during different seasons of the year. Numerous reports have suggested the constant changes of the FF composition related to the physiological status of the growing follicle [11, 12], the physiological and health conditions of the animal [13–15], and the reproductive seasonality variation [16, 17]. These changes can influence the quality of growing oocytes and their readiness for successful fertilization and subsequent embryo development [18–20]. Moreover, investigating the seasonal variation of equine FF composition may help to better comprehend the mechanisms governing oocyte and follicle maturation, facilitating, therefore, assisted reproductive techniques.

High-throughput technologies (e.g., genomics, metabolomics, and proteomics) allow for in-depth investigations of complex samples such as FF, with potential for new biomarker discoveries, or strategies for intrafollicular treatment. Large-scale proteomics approaches (gel-based and gel-free) were applied to either profile or compare global proteomes of FF in cows [21, 22], humans [23, 24], pigs [25, 26], dogs [27], and horses [28, 29]. Currently, the knowledge regarding equine FF proteome is deficient, and its relationship with fertility in mares is still unknown. Furthermore, the relationship of the equine FF dynamics composition with the reproductive seasonality in horses remains to be determined [4].

The aims of the present study were to (i) use the shotgun (gel-free) approach to evaluate the proteome profiles of equine FF collected from ovarian follicles (30–34 mm in diameter) at different seasons of the year (spring anovulatory or SAN, spring ovulatory or SOV, summer or SUM, and fall ovulatory or FOV); and (ii) apply comparative bioinformatics analyses to identify potential regulatory network differences.

## Methods

### Animals

Seventeen Quarter horse mares, 8 to 14 years old and weighing 400 to 600 kg, were housed on pasture in the northern hemisphere (37° 42′ 37.53“ N, 89° 13’ 9.50” W),

under natural light conditions, with free access to fresh water and trace-mineralized salt. Animals were handled in accordance with the US Government Principles for the Utilization and Care of Vertebrate Animals Used in Testing, Research, and Training (https://grants.nih.Gov /grants/olaw /references/phspol.htm #US GovPrinciples). This study was approved by the Institutional Animal Care and Use Committee (IACUC) of Southern Illinois University.

### Ultrasonographic examination and seasonal groups

Follicular fluids were collected from dominant growing follicles during various seasons of the same year: – *March*, as Spring Anestrus (SAN) representing the transition season when dominant anovulatory follicles are found after the deep anestrus season and before the spring ovulatory season; − *Between April and May*, as Spring Ovulatory (SOV) representing the beginning of the ovulatory season, with regular cycling of mares; − *Between June and July*, as Summer (SUM) representing the middle of the ovulatory season, with maximum of ovarian cyclic activity expected; − and *September*, as Fall Ovulatory (FOV) representing the final period of the ovulatory season befor the transition to the fall anovulatory and deep anestrus seasons. In all four seasons, follicles ≥6 mm in diameter were ablated, as previously described [30], to induce a new follicular wave, allowing, therefore, proper tracking of growing/healthy follicles. During the SAN season, after follicle ablation, follicles of the new induced wave were daily tracked using an ultrasound machine (Aloka SSD-900; Aloka Co, Ltd., Wallingford, CT, USA) equipped with a multi-frequency 5–10 MHz linear array transducer (Aloka UST-5821–7.5). Samples of FF (*n* = 6 follicles) were collected when the follicles reached 30–34 mm in diameter. During SOV, SUM, and FOV, mares were monitored daily with ultrasonography until ovulation; thereafter, follicle ablation was performed on day 10–12 after ovulation (day 0 = ovulation) and follicle tracking of the new induced wave was performed daily to collect FF when a dominant follicle reached 30–34 mm in diameter. Samples of FF were aspirated during SOV, SUM, and FOV seasons (*n* = 6, 6, and 12 follicles, respectively). In all seasons, the presence of uterine edema (estrus-like) and the absence of a corpus luteum detected through ultrasonography at the moment of FF collection did qualify the animal for the procedure.

### Follicular fluid collection

Samples of FF were collected using transvaginal ultrasound-guided follicle aspiration as recently reported [10]. Samples were immediately centrifuged at 1600 x g (10 min at 4 °C), followed by a second centrifugation at 3200 x g (15 min at 4 °C) of resulting supernatants. Only clear FF samples, without any visible trace of blood contamination (presence of red blood cells) were stored at − 80 °C until analyses.

### Electrophoresis of follicular-fluid proteins

Optimal isolation of frozen-thawed equine FF proteins was tested through various equine FF:Acetone:Trichloroacetic Acid (TCA) mixture ratios (5:4:1, 1:4:0, and 1.7:3.3:0). Mixtures were incubated (overnight, − 20 °C), centrifuged (9500 g, 10 min, 4 °C), and supernatants were discarded. Cold acetone (1 ml, kept at − 20 °C) was added to each pellet and sample mixtures were vortexed (20 min), centrifuged (9500 g, 10 min, 4 °C), and resulting supernatants (acetone) were discarded. After three repetitions, pellets were dried under the fume hood and resuspended in the nanopure water. All protein samples were subjected to albumin depletion according to the manufacturer’s instruction (ProteoExtract Albumin removal kit; Calbiochem EMD Biosciences, Darmstadt, Germany). Depleted protein samples were mixed with acetone in a 1:4 ratio (*v*/v - FF:Acetone), precipitated overnight at 4 °C, and washed twice with acetone by successive centrifugations (9500 g, 10 min, 4 °C). Final protein samples were quantified (NanoDrop spectrophotometer; Thermo Scientific, Grand Island, NY, USA) and aliquots of each sample were mixed with sample buffer and loaded into wells of a 4–12% sodium dodecyl sulfate polyacrylamide gel electrophoresis (SDS-PAGE). Gels were run as previously described [31], followed by staining with Coomassie blue R-250 reagent to visualize the protein bands.

### Liquid chromatography-mass spectrometry (LC-MS) analysis of follicular-fluid proteins

Extracted FF proteins of each mare were determined (NanoDrop spectrophotometer, ThermoFisher Scientific) and equal amounts of proteins of two to four mares were pooled for each season (SAN, SOV, SUM, and FOV). For proteomic analyses, three independent pools (100 μg protein each) were constituted for each season. Pooled samples were precipitated overnight with 100% acetone (1:5 ratio), washed two times with 100% acetone, air-dried, and stored at − 20 °C. Prior to in-solution digestion, protein precipitates were dissolved in 100 μl of 100 mM ammonium/5% acetonitrile, reduced with 1/10 volume of 100 mM dithiothreitol (DTT) for 15 min at 65 °C, and alkylated with 1/10 volume of 10 mM iodoacetamide (IAA) for 30 min at room temperature in dark. Digestion was carried out with Trypsin/Lys-C Mix (Promega, Madison, WI) at 37 °C overnight. Samples were freeze-dried and protein tryptic digest was resuspended in 0.1% (*v*/v) formic acid, 2.0% (v/v) acetonitrile. Aliquots of peptides representing two micrograms of protein were subjected to LC-MS analysis as described previously [32]. Briefly, peptides were separated using Ultimate 3000 HPLC system and reversed phase C18, 75 μm × 150 mm column (both Thermo Fisher Scientific), via 170 min long, nonlinear, constant flow (0.3 μl/ml) gradient of acetonitrile (in 0.1% formic acid) as follows: 2–55% for 125 min, 95% for 15 min, 2% for 30 min. Raw mass spectral data were collected by LTQ-Orbitrap Velos mass spectrometer (Thermo Fisher Scientific) working in the result dependent acquisition (RDA) mode of 18 scan events: one MS scan (m/z range: 300–1700) followed by 17 MSMS scans for the 17 most intense ions detected in MS scan, with dynamics exclusion allowed.

### Protein identification and bioinformatics analyses

The raw files were searched using the SEQUEST algorithm of the Proteome Discoverer 1.1.0 software (Thermo Fisher Scientific) as described previously [33]. Variable modifications were considered as follows: cysteine carbamidomethylation (+ 57.021), methionine oxidation (+ 15.995), methionine dioxidation (+ 31.990). The spectral data were matched against target and decoy databases to allow for calculation of false discovery rates (FDR). The NCBI (www.ncbi.nlm.nih.gov) *Equus caballus* taxonomy referenced protein database (36,108 entries as of August 2017) served as the target database, while its reversed copy (created automatically by the software) served as a decoy database. The search results were filtered by FDR < 1% for high-confidence protein identification. Proteins were functionally annotated (Gene ontology or GO, Enrichment, KEGG pathway, and protein-protein interactions) using the online tools of Agbase (http://agbase.arizona.edu/), DAVID (Database for Annotation, Visualization and Integrated Discovery; DAVID Bioinformatics Resources 6.8; https://david.ncifcrf.gov/home.jsp), and STRING (https://string-db.org/cgi/input.pl?sessionId=LyvanBxDO3QN&input_page_show_search=on) using the default settings.

## Results

### Sample preparation prior to proteomic analysis

**Fig. 1:**
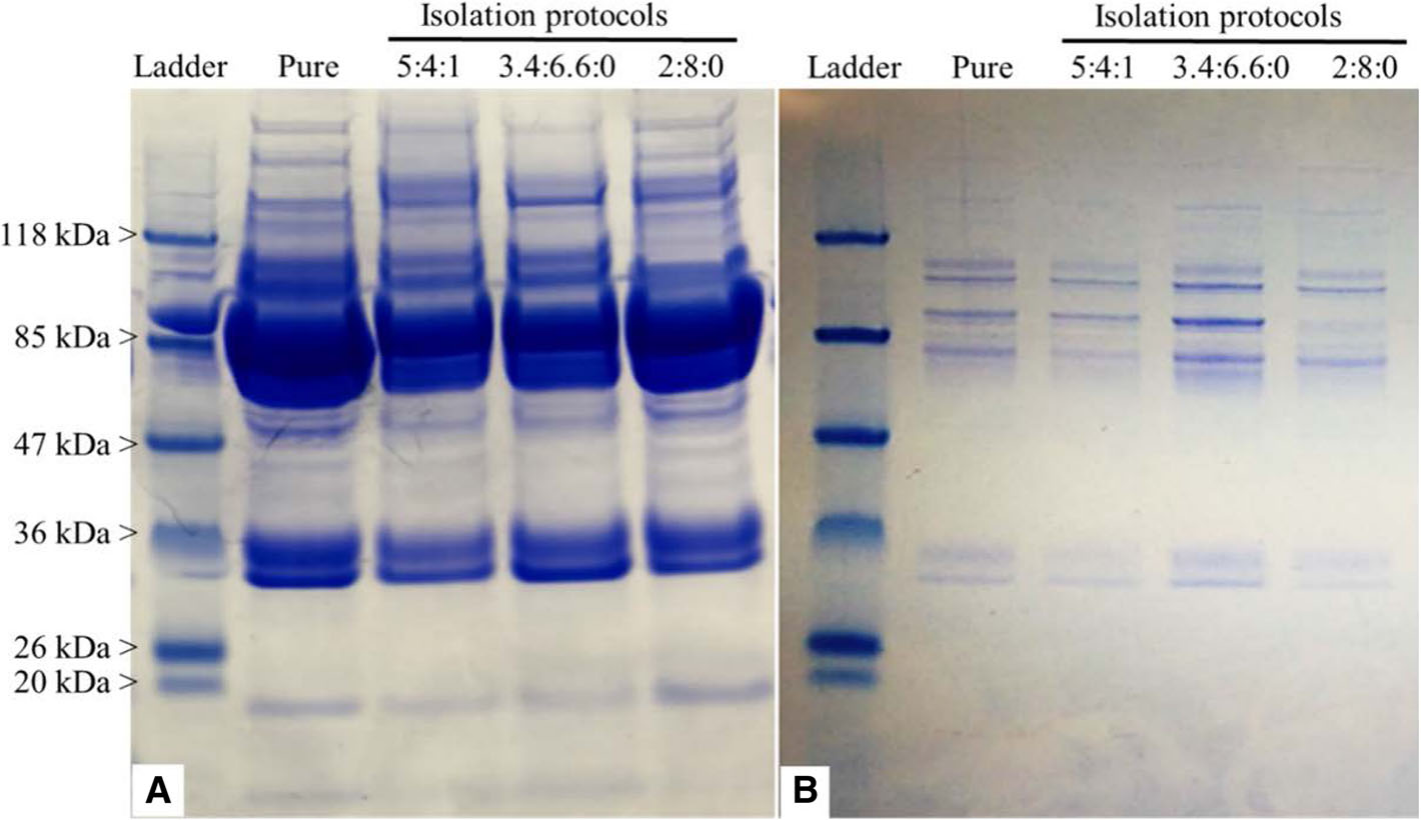
Follicular fluid (FF) protein isolation through combined precipitation and depletion approach. Representative gel electrophoresis of equine FF submitted to four different Acetone-TCA-based protein precipitation protocols (**a**), followed by albumin depletion (**b**) are shown. Gels were stained with Coomassie blue to visualize the protein bands, showing decreased protein amounts following both precipitation and depletion. Utilization of pure FF revealed higher protein recovery following depletion. Extraction protocols (5:4:1, 3.4:6.6:0, and 2:8:0) corresponded to FF:Acetone:TCA, respectively

The protein concentrations of pure FF derived from all seasons (SAN, SOV, SUM, and FOV) averaged 39.2 ± 0.4, 38 ± 0.3, 38.3 ± 0.4, and 39 ± 0.4 μg/μl, respectively. The use of pure FF samples (33.2 ± 0.4 μg/μl) for protein precipitation tests (in 5:4:1, 1.7:3.3:0, and 1:4:0 solvent ratios) resulted in decreased protein concentrations (5.8 ± 0.1, 7.9 ± 0.2, and 22.8 ± 0.4 μg/μl, respectively), while the additional albumin depletion procedure led to lesser protein concentrations in all tested FF groups (0.1 ± 0.01, 0.14 ± 0.01, and 0.41 ± 0.02 μg/μl for 5:4:1, 1.7:3.3:0, and 1:4:0 solvent ratios, respectively). Representative electrophoresis gels of both precipitated (Fig. 1a) and precipitated/depleted proteins (Fig. 1b) indicate comparable protein profiles across samples. Although the depletion of pure FF samples (33.2 ± 0.4 μg/μl) produced lower protein concentrations (0.59 ± 0.2 μg/μl), the recovery rate and gel electrophoresis protein profiles were satisfactory for further proteomic analysis.

### Total proteins identified

**Fig. 2:**
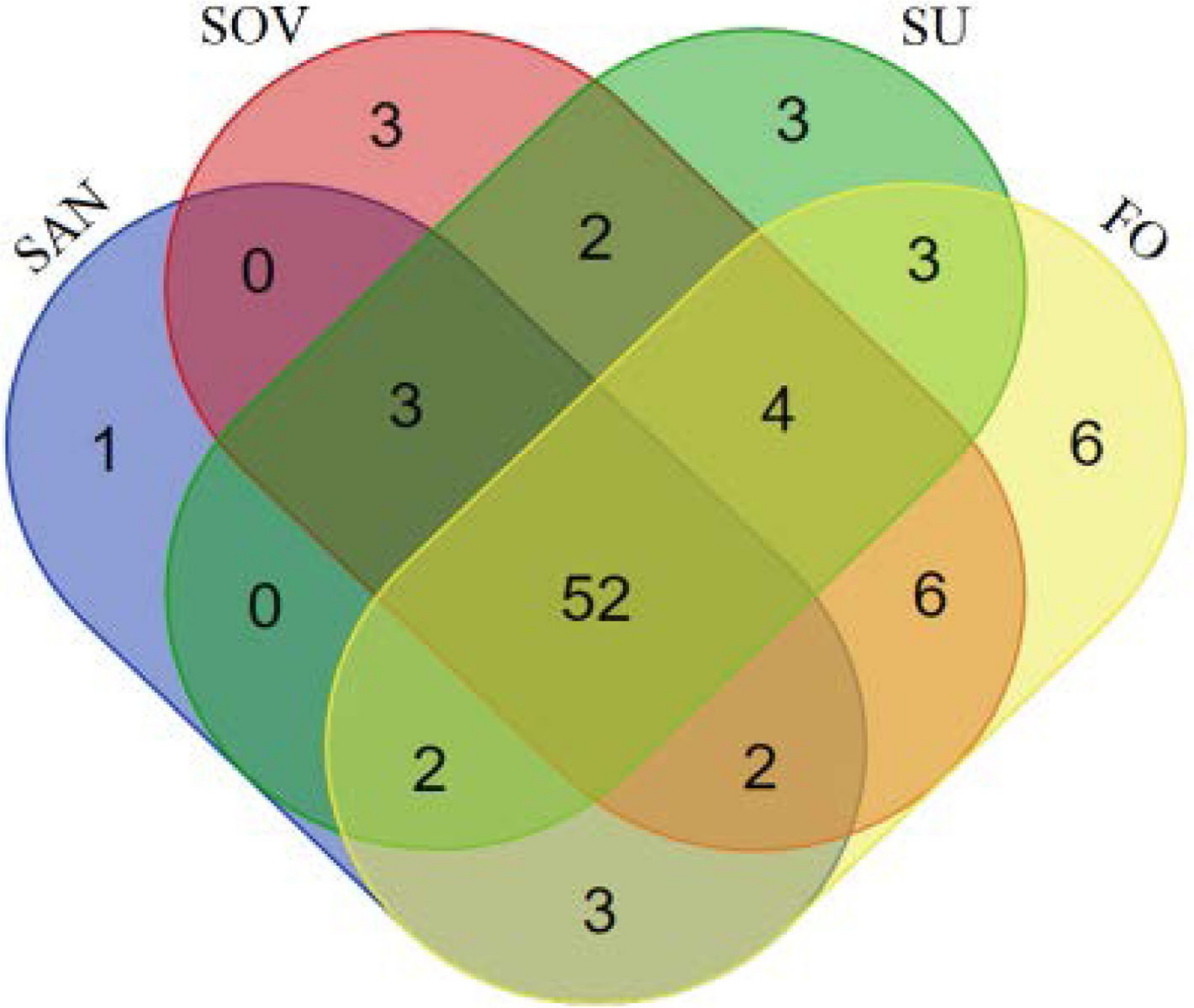
Venn diagram representation of proteins identified in equine follicular fluid across seasons. Spring anovulatory (SAN), spring ovulatory (SOV), summer (SUM or SU) and fall ovulatory (FOV or FO) seasons

All identified proteins are summarized (Table 1). The totals of 63, 72, 69, and 78 proteins were identified with high confidence (FDR < 1%) in SAN, SOV, SUM, and FOV samples, respectively. Approximately 87% of proteins were annotated with the NCBI-non redundant database, and 13% with ENSEMBL. The Venn diagram (http://bioinformatics.psb.ugent.be/webtools/Venn/) indicates 52 proteins shared across all seasons, 25 proteins detected in two or three different seasons, and 13 unique proteins identified in a specific season (one for SAN, three for SOV, three for SUM, and six for FOV; Fig. 2). Overall, a total of 90 proteins were detected in the FF samples across all seasons. Proteins found in each intersection of the Venn diagram are listed in a textual output (Table 2**)**, and all seasonal proteome datasets with full protein annotations are provided as supplementary data (Additional file 1: Table S1).

**Table 1:** Seasonal variation of equine follicular fluid proteome

Reproductive seasons	Number of detected proteins			
	N	NCBI annotated (%)		ENSEMBL annotated (%)
		Partially	Fully	
Spring anovulatory (SAN)	63	42 (66.7)	12 (19.0)	9 (14.3)
Spring ovulatory (SOV)	72	48 (66.7)	14 (19.4)	10 (13.9)
Summer (SUM)	69	47 (68.1)	13 (18.8)	9 (13.0)
Fall ovulatory (FOV)	78	49 (62.8)	18 (23.1)	11 (14.1)

**Table 2:** Equine follicular fluid proteins distribution across four reproductive seasons

Reproductive seasons	Number of unique proteins	NCBI/ENSEMBL accession numbers
SAN/SOV/SUM/FOV	52	ENSECAP00000007499 XP_001498388 XP_001489154 XP_003365492 XP_001504386 XP_014588282 XP_001502426 NP_001075422 XP_014596181 XP_003363176 XP_001500552 NP_001075379 XP_005605480 XP_005601929 XP_005602671 XP_001499389 XP_005611649 XP_005612174 NP_001304178 ENSECAP00000010483 XP_014594947 XP_001488384 XP_014593946 XP_005600608 XP_001497860 NP_001075415 XP_001916967 XP_001492943 NP_001075972 XP_001499173 XP_001502503 XP_001496277 XP_001492602 ENSECAP00000017379 NP_001075413 ENSECAP00000012479 ENSECAP00000014609 ENSECAP00000012399 XP_001915589 XP_001489797 XP_001503846 XP_003364583 XP_001490892 ENSECAP00000013972 XP_001914833 XP_014585351 XP_001492576 XP_001489400 XP_001495232 XP_014593950 ENSECAP00000009723 XP_001504173
SAN/SOV/SUM	3	NP_001333128 XP_001488181 XP_001488234
SAN/SOV/FOV	2	NP_001075419 NP_001093235
SAN/SUM/FOV	2	XP_005607860 XP_014593981
SOV/SUM/FOV	4	NSECAP00000018005 NP_001137426 NP_001075389 XP_001501882
SAN/FOV	3	NP_001333146 NP_001333133 ENSECAP00000017139
SOV/SUM	2	NP_001333066 XP_005605484
SOV/FOV	6	XP_014591249 NP_001075249 XP_005601424 XP_001491754 XP_001493453 NP_001075420
SUM/FOV	3	NP_001075378 XP_001496318 NP_001108005
SAN	1	NP_001333005
SOV	3	XP_001504484 XP_001504447 ENSECAP00000013036
SUM	3	XP_005599640 XP_005605481 NP_001271464 XP_001917127
FOV	6	ENSECAP00000012950 NP_001157490 XP_001499312 NP_001108630 XP_001492582 XP_014592983

There were no shared proteins among the following groups: SAN/SOV and SAN/SU. *SAN* spring anovulatory, *SOV* spring ovulatory, *SUM* summer, *FOV* fall ovulatory

### Functional classification, protein enrichment, and pathways analyses

**Fig. 3:**
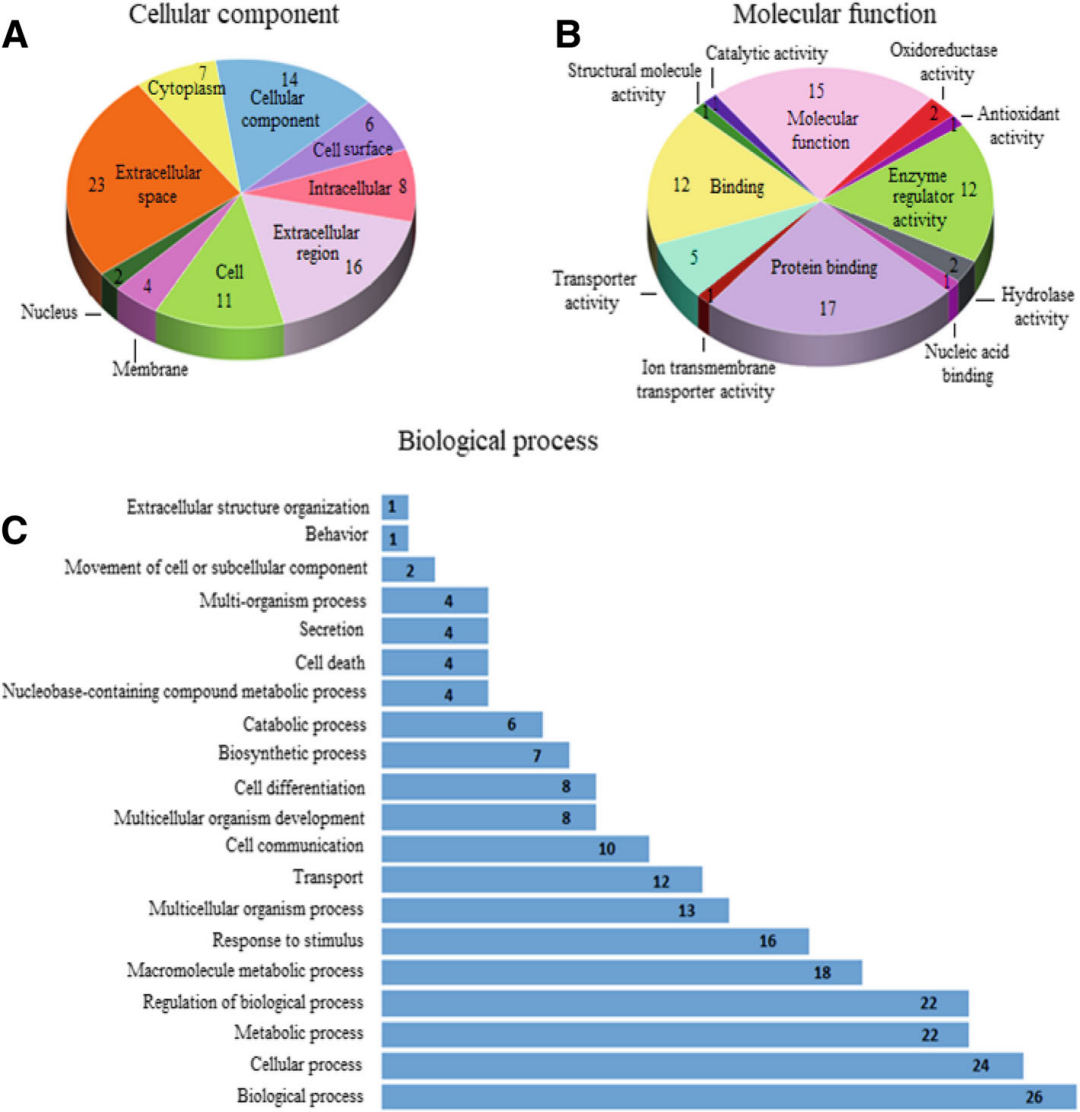
Functional categorization of proteins shared across all seasons. Distribution of total protein per gene ontology (GO) terms in the Cellular component (**a**), Molecular function (**b**), and Biological process (**c**) categories

For functional classification, GO annotation was available for 88.5 to 91.7% of identified proteins across the season datasets. Proteins were classified into three GO categories as cellular components (CC), molecular functions (MF), and biological processes (BP). Regardless of season, proteins were distributed within 9–10, 12, and 20 GO terms associated with CC, MF, and BP, respectively. The functional categorization of shared proteins and the observed quantitative variations in GO terms constituting each functional category are shown (Fig. 3).

Irrespective of the season, GO terms associated with extracellular components (space, region, cell surface, membrane, and proteinaceous) represented approximately 54% of the total annotations within the CC category (Table 3). These specific GO terms were 6x to 9x enriched (*P* < 10^− 5^, FDR < 0.01). Other GO names such as “blood microparticle” and “fibrinogen complex” were significantly enriched (>104x; P < 10^− 4^, FDR < 0.01) in our datasets. Similarly, the GO terms associated with binding (binding, protein, and nucleic acid) were highly represented (~ 49%) within the MF category (Table 4), and only the “serine-type endopeptidase inhibitor activity” GO term revealed significant enrichment across all seasons (46-63x; *P* < 10^− 16^, FDR < 0.01). Nonetheless, several GO terms associated with binding (e.g., heparin, cholesterol, phosphatidylcholine, and copper ion) and enzymatic activity (e.g., cholesterol transporter, phosphatidylcholine-sterol O-acyltransferase activator, structural molecule, cysteine-type endopeptidase inhibitor) were significantly enriched (>6x; *P* < 0.05), but with higher FDR (> 0.01). As for the BP category across seasons, approximately 70, 8, 5, and 4% of total annotations were respectively associated with “processes”, “response to stimulus”, “transport”, and “cell communication” (Table 5). Meanwhile, the “acute-phase response”, “fibrinolysis”, and “positive regulation of cholesterol esterification” GO terms were significantly enriched in all seasonal samples (>62x; *P* < 10^− 3^, FDR < 0.01), and the “blood coagulation” GO term was substantially enriched in the SUM samples only (36x; P < 10^− 3^; FDR < 0.01). Finally, the “complement and coagulation cascades” KEGG pathway was significantly enriched (50-54x; P < 10^− 3^; FDR < 0.01), regardless of the reproductive season.

**Table 3:** Cellular component of equine follicular fluid proteins across four different reproductive seasons

GO terms / GO names	Reproductive seasons				Shared
	SAN n (%)	SOV n (%)	SUM n (%)	FOV n (%)	proteins n (%)
GO:0005615 / Extracellular space	25 (24.0)	29 (23.8)	29 (26.9)	32 (25.4)	23 (25.3)
GO:0005575 / Cellular component	17 (16.3)	21 (17.2)	18 (16.7)	22 (17.5)	14 (15.4)
GO:0005576 / Extracellular region	17 (16.3)	19 (15.6)	19 (17.6)	21 (16.7)	16 (17.6)
GO:0005623 / Cell	14 (13.5)	17 (13.9)	13 (12.0)	16 (12.7)	11 (12.1)
GO:0005622 / Intracellular	9 (8.7)	12 (9.8)	10 (9.3)	11 (8.7)	8 (8.8)
GO:0005737 / Cytoplasm	7 (6.7)	8 (6.6)	7 (6.5)	8 (6.3)	7 (7.7)
GO:0009986 / Cell surface	6 (5.8)	6 (4.9)	6 (5.6)	6 (4.8)	6 (6.6)
GO:0016020 / Membrane	6 (5.8)	6 (4.9)	4 (3.7)	6 (4.8)	4 (4.4)
GO:0005634 / Nucleus	3 (2.9)	3 (2.5)	2 (1.9)	3 (2.4)	2 (2.2)
GO:0005578 / Proteinaceous extracellular matrix	–	1 (0.8)	–	1 (0.8)	–
Total annotations	104 (100)	122 (100)	108 (100)	126 (100)	91 (100)

*SAN* spring anovulatory, *SOV* spring ovulatory, *SUM* summer, *FOV* fall ovulatory

**Table 4:** Molecular function of equine follicular fluid proteins across four different reproductive seasons

GO terms / GO names	Reproductive seasons				Shared
	SAN n (%)	SOV n (%)	SUM n (%)	FOV n (%)	proteins n (%)
GO:0005488 / Binding	22 (26.2)	25 (27.2)	25 (27.2)	29 (27.9)	21 (27.6)
GO:0005515 / Protein binding	18 (19.6)	18 (19.6)	19 (20.7)	20 (19.2)	17 (19.0)
GO:0003674 / Molecular function	16 (19.6)	16 (17.4)	17 (18.5)	20 (19.2)	15 (18.1)
GO:0030234 / Enzyme regulator activity	12 (14.3)	12 (13.0)	13 (14.1)	13 (12.5)	12 (11.2)
GO:0005215 / Transporter activity	5 (6.0)	6 (6.5)	5 (5.4)	6 (5.8)	5 (5.2)
GO:0016787 / Hydrolase activity	2 (2.4)	4 (4.3)	2 (2.2)	4 (3.8)	2 (3.4)
GO:0005198 / Structural molecule activity	2 (2.4)	4 (4.3)	3 (3.3)	3 (2.9)	1 (4.3)
GO:0016491 / Oxidoreductase activity	2 (2.4)	2 (2.2)	3 (3.3)	3 (2.9)	2 (1.7)
GO:0003676 / Nucleic acid binding	2 (2.4)	1 (1.1)	2 (2.2)	2 (1.9)	1 (3.4)
GO:0003824 / Catalytic activity	1 (1.2)	2 (2.2)	1 (1.1)	2 (1.9)	1 (2.6)
GO:0015075 / Ion transmembrane transporter activity	1 (1.2)	1 (1.1)	1 (1.1)	1 (1.0)	1 (0.9)
GO:0016209 / Antioxidant activity	1 (1.2)	1 (1.1)	1 (1.1)	1 (1.0)	1 (0.9)
Total annotations	84 (100)	92 (100)	92 (100)	104 (100)	79 (100)

*SAN* spring anovulatory, *SOV* spring ovulatory, *SUM* summer, *FOV* fall ovulatory

**Table 5:** Biological process of equine follicular fluid proteins across four different reproductive seasons

GO terms / GO names	Reproductive seasons				Shared
	SAN n (%)	SOV n (%)	SUM n (%)	FOV n (%)	proteins n (%)
GO:0008150 / Biological process	31 (13.2)	35 (13.8)	32 (12.7)	40 (13.7)	26 (12.3)
GO:0009987 / Cellular process	26 (11.1)	29 (11.5)	28 (11.2)	32 (11.0)	24 (11.3)
GO:0050789 / Regulation of biological process	25 (10.7)	28 (11.1)	27 (10.8)	30 (10.3)	22 (10.4)
GO:0008152 / Metabolic process	24 (10.3)	25 (9.9)	26 (10.4)	30 (10.3)	22 (10.4)
GO:0043170 / Macromolecule metabolic process	20 (8.5)	20 (7.9)	20 (8.0)	24 (8.2)	18 (8.5)
GO:0050896 / Response to stimulus	19 (8.1)	20 (7.9)	19 (7.6)	25 (8.6)	16 (7.5)
GO:0032501 / Multicellular organism process	14 (6.0)	17 (6.7)	16 (6.4)	18 (6.2)	13 (6.1)
GO:0006810 / Transport	12 (5.1)	14 (5.5)	13 (5.2)	16 (5.5)	12 (5.7)
GO:0007154 / Cell communication	10 (4.3)	10 (4.0)	11 (4.4)	11 (3.8)	10 (4.7)
GO:0007275 / Multicellular organism development	9 (3.8)	10 (4.0)	10 (4.0)	11 (3.8)	8 (3.8)
GO:0030154 / Cell differentiation	9 (3.8)	9 (3.6)	10 (4.0)	11 (3.8)	8 (2.8)
GO:0009058 / Biosynthetic process	8 (3.4)	7 (2.8)	8 (3.2)	9 (3.1)	7 (3.3)
GO:0009056 / Catabolic process	6 (2.6)	7 (2.8)	6 (2.4)	8 (2.7)	6 (2.8)
GO:0006139 / Nucleobase-containing compound metabolic process	5 (2.1)	4 (1.6)	5 (2.0)	6 (2.1)	4 (1.9)
GO:0008219 / Cell Death	4 (1.7)	4 (1.6)	5 (2.0)	5 (1.7)	4 (1.9)
GO:0046903 / Secretion	4 (1.7)	4 (1.6)	5 (2.0)	5 (1.7)	4 (1.9)
GO:0051704 / Multi-organism process	4 (1.7)	4 (1.6)	6 (2.4)	5 (1.7)	4 (1.9)
GO:0006928 / Movement of cell or subcellular component	2 (0.9)	3 (1.2)	2 (0.8)	2 (0.7)	2 (0.9)
GO:0043062 / Extracellular structure organization	1 (0.4)	1 (0.4)	1 (0.4)	2 (0.7)	1 (0.5)
GO:0007610 / Behavior	1 (0.4)	1 (0.4)	1 (0.4)	1 (0.3)	1 (0.5)
Total annotations	234 (100)	253 (100)	251 (100)	291 (100)	212 (100)

*SAN* spring anovulatory, *SOV* spring ovulatory, *SUM* summer, *FOV* fall ovulatory

### Comparison between reproductive seasons

1Qualitative and quantitative differences in GO terms constituting each functional category were found across seasons (Tables 3-5). GO terms associated with “extracellular space” (in CC category) and “protein binding” (in MF category) increased in SUM compared to other seasonal groups. In contrast, GO terms associated with “membrane” and “nucleus” (in CC category), “transporter activity” (in MF category), and “response to stimulus” (in BP category) were decreased in SUM. Moreover, the proportions of annotations associated with “transporter activity” in MF and “response to stimulus” in BP were higher in SOV and FOV, respectively, compared to other seasons. Finally, GO terms associated with “intracellular” (in CC category), “binding”, “hydrolase activity”, and “structural molecule activity” (in MF category), and “biological process”, and “multicellular organism process” (in BP category) were lower in SAN compared to SOV.

### Protein-protein interaction (PPI) network analyses

**Fig. 4:**
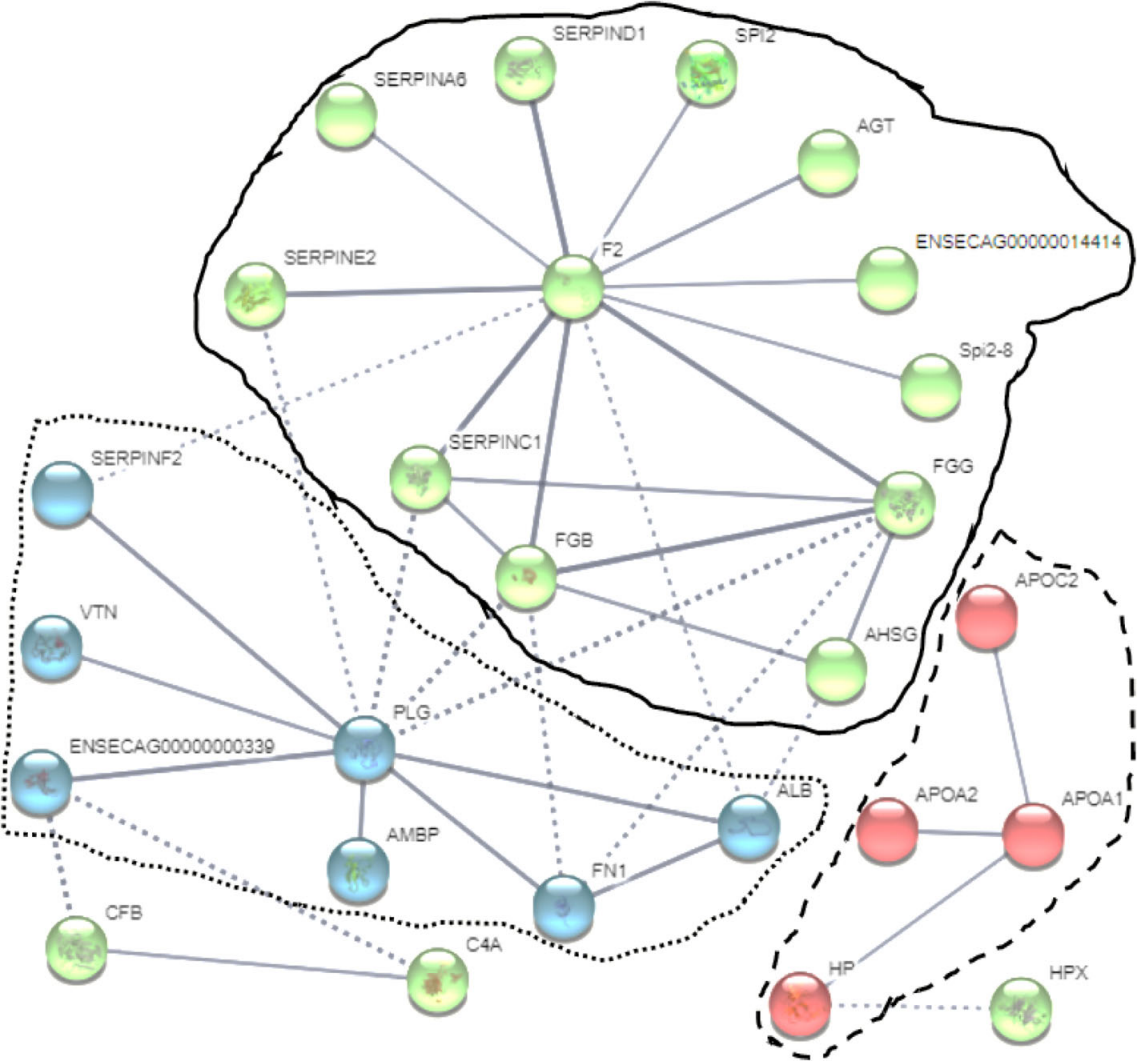
Protein-to-Protein Interaction network of proteins shared across all seasons

The PPI analysis was performed for each season, including the shared protein dataset. For each dataset, three major PPI K-means clustering were obtained with high confidence interaction score (> 0.7) and significant PPI enrichment (*P* < 10^− 16^). A representative PPI network generated from shared dataset is shown (Fig. 4). The three main clusters (circles) and related key proteins having higher numbers of interactions are indicated. Cluster 1 (green in Fig. 4) revealed F2 protein (or prothrombin) with the greatest interactions in SAN (*n* = 12), SOV (*n* = 14), FOV (*n* = 13), and shared (n = 12) protein datasets, while the combination of F2 (n = 13) with PLG (plasminogen, *n* = 11) appeared as the main players in the SUM dataset. In cluster 2 (blue in Fig. 4), plasminogen had the highest number of interactions in SAN and shared datasets (*n* = 10), while the combination of both plasminogen (n = 10) and ENSECAG00000000339 (complement C3) with four and five interactions may have important roles during SOV and FOV seasons, respectively. Contrarily in the SUM dataset, the F12 (coagulation factor XII) protein appeared as the main player with 12 interactions. Finally, the cluster 3 (red in Fig. 4) revealed APOA1 (apolipoprotein 1) protein as the key player in all seasons.

## Discussion

The current study uses a gel-free technique to provide unique proteomic datasets of equine FF of dominant growing follicles (30–34 mm in diameter) during the SAN, SOV, SUM, and FOV seasons. The existence of seasonal proteins in a similar follicle class, reported herein for the first time, suggests potential critical roles of FF proteins during the folliculogenesis and maybe oogenesis in the equine species. Likewise, various biological functions, protein enrichment, and protein interaction networks are reported to have been influenced by the seasonal variations.

### Follicular fluid proteins isolation

Combination of procedures, such as protein precipitation and depletion of high-abundant proteins, are routinely used to enhance the quality of starting samples for proteomic analyses [28, 34, 35]. In this study, all tested precipitation protocols (FF:Acetone:TCA ratio of 5:4:1, 1:4:0, and 1.7:3.3:0) resulted in expected lower protein concentrations (30 to 82% losses) that were exacerbated by a further depletion of high-abundance serum protein (about 99% losses). Interestingly, the electrophoretic profiles of protein samples were generally comparable, regardless of the procedure. Crude equine FF samples maintained the highest protein concentrations following depletion, with only 30% loss, from 33.2 ± 0.4 μg/μl to 22.8 ± 0.4 μg/μl. Thereafter, the 1:4:0 precipitation ratio appeared the most suitable with lesser protein loss, which was consistent with a previous report Santa et al. [36].

### Proteome description

The gel-free LC-MS proteomics has been successfully used in previous studies of FF of stock animals [21, 23, 26]. The present study applied strict filters (FDR < 1%, and the minimum of two unique peptides per protein) to obtain proteins with high confidence identification, which may explain the slightly lower number of detected proteins (90 vs. 113) when compared to available FF mare proteome [28]. In addition, the proteome dataset of the current study contains fewer proteins in comparison to other monovular species such as humans (158 to 1079; [24, 37–40]), and dairy cattle (113 to 219; [15, 21]). Nonetheless, the aforementioned proteomic studies were generally performed with FF samples obtained from follicles of different sizes and unknown physiological statuses (i.e., growing and regressing follicles), under different technical approaches (e.g., gel-based or gel-free) and protein call stringencies (e.g., false discovery rate and peptides). The full annotation (87% with NCBI and 13% with ENSEMBL) of all detected proteins offers opportunities for in-depth investigations, such as the dynamic composition of the equine FF proteome and the relationship with oocyte quality. To the best of our knowledge, this is the first study providing essential clues of the FF proteome variations to enable a further understanding of the impact of different seasons on fertility of the mare, and maybe of other livestock.

### Proteins specifics to seasons

Among the 90 proteins, a core set of 52 was detected across all seasons. It is expected that these proteins may have essential roles during folliculogenesis [39] and oogenesis [2] processes, as previously reported in humans. In contrast, the examined seasons were characterized by subsets of proteins that may serve as potential biomarkers of seasonal fertility in mares. For instance, the BPI (Bactericidal/Permeability-Increasing) fold-contain Family A member 2 (BPIFA2) precursor was found only in SOV, SUM, and FOV (ovulatory seasons). The BPI is an endogenous antibiotic protein that belongs to the family of mammalian lipopolysaccharide (LPS)-binding and lipid transport protein. The BPIFA2 is known to have a role in the innate immune responses and was reported to inhibit the formation of biofilm by pathogenic gram-negative bacteria in the respiratory tract [41]. In this regard, although the function for BPI in FF is still unknown, the presence of BPIFA2 during the ovulatory seasons may be important to protect the female genital tract (e.g., oviduct). Also, few reports have found that BPI is expressed in the testis and epididymis of mice and appears to take part in the process of gamete interactions [42, 43].

In contrast, keratin-10 was detected in SAN samples only and may, therefore, be associated with the non-ovulatory seasons. Although keratin is considered a common contaminant in proteomic studies, the keratin-10 family member has been reported as a negative modulator of cell cycle progression throughout the Phospho-Inositol 3 kinase (PI3 kinase) signal transduction pathway [44]. Numerous studies have reported the participation of PI3 kinase in the follicle-stimulating hormone or progesterone-induced meiotic oocyte maturation in Xenopus [45, 46] and mouse [47, 48]. In the present study, eight keratin-like family members were present in different intersections of the Venn diagram, and their specific roles in the acquisition of the oocyte developmental competence remain to be unfolded.

### Functional analyses

In this study, the interpretation of bioinformatics analyses focused only on proteins exhibiting thresholds of significance that were lower than 1% in both Benjamini-Hochberg and FDR analyses. About half of the protein annotations belonged to the Extracellular GO term, and only 10% of the total annotations were attributed to Intracellular localization, regardless of the proteome dataset. This distribution is expected, given the composition of the FF, known to contain secretions of follicle cells and blood plasma exudates. Thus, proteins attributed to Intracellular regions may be residues of the various catabolic processes and/or cell breakdown (apoptosis) of follicle cells (granulosa cells) that occur throughout the follicle growth [39, 49, 50]. Protein distributions within the present FF datasets are in agreement with previous reports in other species [24, 51, 52], but differ from the uniquely available report in horses [28], indicating 83% of protein annotations within the Extracellular region and 17% Intracellular. This difference may be due to either the mare breeds (Welsh pony vs. Quarter horse in the current study) or their proteome dataset generated from the combination of distinct follicle physiological stages.

Regarding protein functions, approximately 49% of the total annotations belonged to binding (protein binding and nucleic acid binding GO terms), and 32% corresponded to other cellular and molecular activities. This specific distribution is in agreement with previous studies in humans [24, 51], and reflects the participation of FF proteins in a variety of physiological functions associated with follicle and oocyte growth. In this study, several proteins belonging to the serine-type endopeptidase inhibitor activity, binding (heparin, cholesterol, and copper ion), and enzyme transporter (cholesterol) were significantly enriched across all seasons; those proteins have also been detected in other mono-ovulatory species [28, 52].

Proteins associated with inflammatory responses (immune system, coagulation, acute phase response signaling), a FF signature across studies and species such as humans [53], goats [52], cattle [21], and horses [28], were significantly enriched in our datasets. These proteins may participate in cascades of immune and coagulation formation (fibrin) /inhibition (anti-thrombin) responses having vital roles in follicle growth and oocyte transfer to the oviduct following ovulation. The anticoagulation function of the FF has been revealed to be essential during follicle growth and rupture [54]; moreover, in all of our datasets (SAN, SOV, SUM, and FOV), a significant enrichment in proteins associated with the coagulation cascade was noticed.

The functional categorization indicated a higher proportion of proteins associated with the “Extracellular space” GO name during the SUM season. The increase in FF protein content found in our study during the SUM season may have been due to an increase in ovarian vascularization/blood flow [9, 55, 56], likely favoring the entry of additional plasma proteins into the follicle. Interestingly, the SAN dataset exhibited lower numbers of proteins associated with “Intracellular” (in CC); “Hydrolase activity”, “Structural molecule activity”, and “Binding” (in MF); and “Biological process” and “Multicellular organism process” (in BP) than that of the SOV dataset. These differences may lead to further understanding of the differences in FF environment of dominant anovulatory versus ovulatory follicles during the SAN and SOV seasons, respectively.

### Protein and pathway enrichments

Proteins associated with the “complement and coagulation cascades” pathway were significantly enriched in all seasons: 25.4% in SAN, 23.6% SOV, 23.2% SUM, and 25.6% FOV. Indeed, the complement system and inflammatory processes regulate follicle development and ovulation [57–59]. Numerous proteins are known to play essential roles during major events of the ovarian follicle [60]. These events involve a variety of proteolytic and metabolic processes that are mediated by several enzymes found in our datasets. Furthermore, the synthesis of some proteins may have been favored by the high number of protease inhibitors found in our study, such as fetuin-B, plasma protease C1 inhibitor, protein Z-dependent protease inhibitor, alpha-1-antiproteinase 2, alpha-1-antichymotrypsin, GDN peptidase inhibitor 7, inter-alpha-trypsin inhibitor heavy chain H1, H2 and H4, and SERPIN for serine-protease inhibitors. Among them, the SERPIN, a superfamily of protease inhibitors [61], are involved in follicle development and may regulate the follicular extracellular matrix remodeling [22]. On the other hand, many other proteins were associated with coagulation cascades. The presence of proteins such as antithrombin-III, alpha macroglobulin, plasminogen, alpha-2-antiplasmin, and fibrinogen indicates their participation in the controlling, modeling, and regulation of the coagulation pathway leading to healthy follicle growth.

### Protein-protein interaction (PPI) networks

**Fig. 5:**
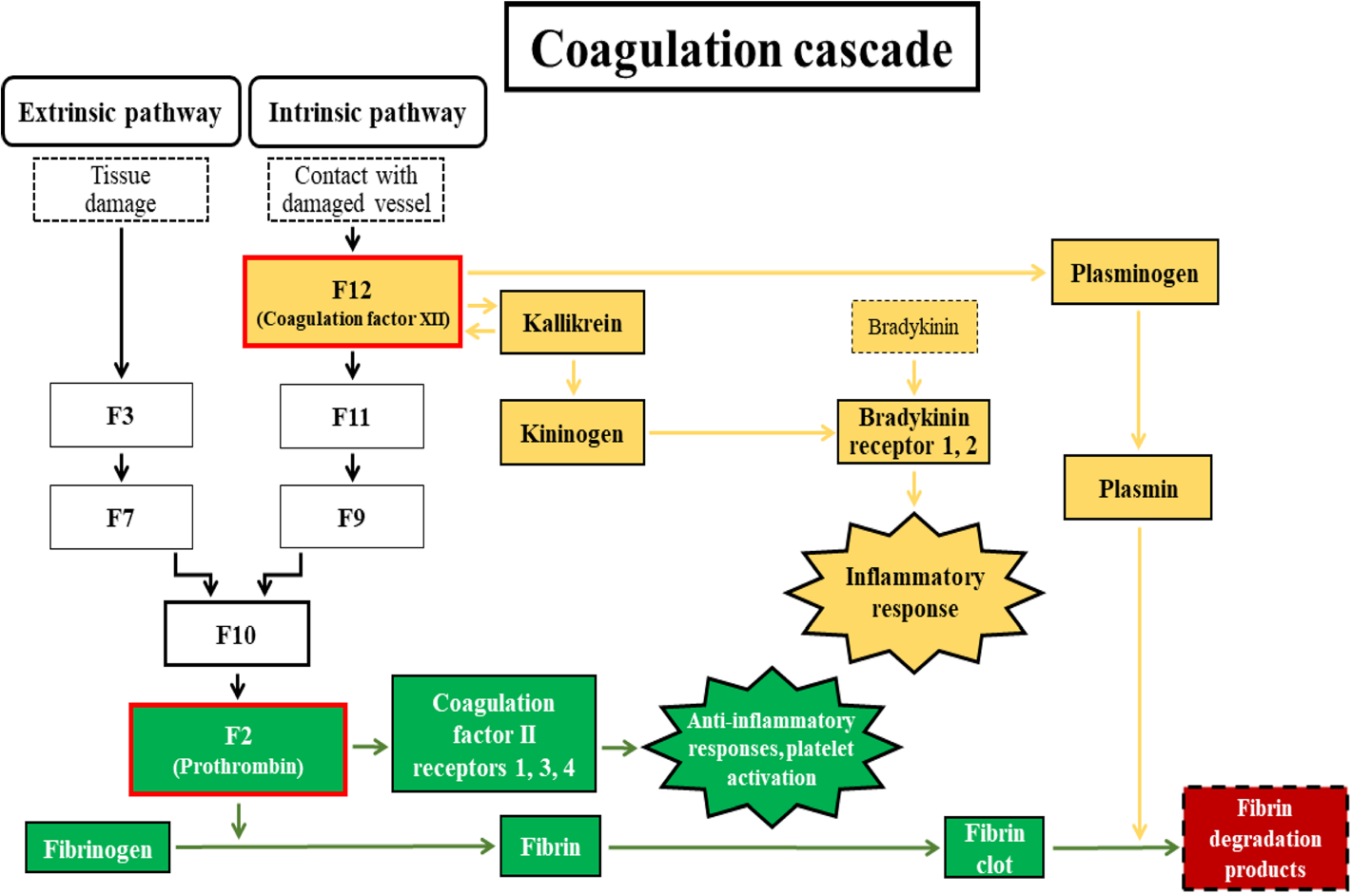
Schematized “coagulation cascade” pathway derived from proteins shared across all seasons. Adapted from KEGG Pathway Database of “Coagulation and Complement Cascade” (map04610; http://www.genome.jp/kegg/pathway.html). This diagram shows the roles of the F12 protein (Coagulation factor XII; yellow color), in the intrinsic pathway and the F2 protein (Prothrombin; green color), in the extrinsic pathway of the cascade, leading to the “fibrin degradation products”

The PPI network information is one of the major fields in systems biology allowing for complex network analyses [62]. The PPI permitted the consolidation of the “coagulation cascade” (Fig. 5) as a main signature of the equine FF, as seen in all datasets (SAN, SOV, SUM, FOV, and shared proteins) and previous reports in various species [28, 52, 53]. Clustering analyses allowed the prediction of the combination of F12 (coagulation factor XII), F2 (prothrombin), and PLG (plasminogen) as the signature of equine FF proteins during SUM, while the F2-PLG-ENSECAG00000000339 (complement C3) combination had higher interactions in both the SOV and FOV seasons. In contrast, both F2 and PLG were mainly found during the SAN season. These observations are significant given the functions of the implicated proteins.

Firstly, prothrombin or F2 is a glycoprotein and an essential component of the blood-clotting mechanism exerting effects through its mature form, thrombin, by interacting with specific receptors (or protease-activated receptors or ThRs) on the granulosa cell membrane [63, 64]. However, its contribution as an anti-inflammatory compound prone to induce hemorrhagic anovulatory follicles during the transitional reproductive seasons remains to be explored. Secondly, the coagulation factor XII or F12, however, is a pro-inflammatory protein interacting with prekallikrein to initiate a cascade of events leading to the release of bradykinin [65], which in turn increases the action of LH, contributes to follicular wall contraction [66], and favors ovulation [66–68]. These observations are supportive of the increased protein-protein interactions of the coagulation factor XII during SUM, having possible roles in the ovulation outcome in mares. Thirdly, the proteolytic factor plasminogen is capable of dissolving fibrin of blood clots and performs essential functions during reproductive processes such as extracellular matrix remodeling, modulating follicular development, corpus luteum formation, and weakening the follicle wall to promote ovulation [39, 53, 69–71]. Lastly, the high level of apolipoprotein-1 (APOA1) participates in the cholesterol and triglyceride transportation, having positive mitogenic and angiogenic effects [72], which is beneficial to follicle development.

In summary, this study describes, for the first time, the proteome profile of the equine FF collected during anovulatory (SAN) and ovulatory (SOV, SUM, and FOV) seasons. Functional analyses revealed differences that may be essential to better characterization of reproductive seasonality in mares. The findings show that SUM follicular fluid proteome of dominant follicles (30–34 mm) differs from other seasons and appears to be characterized by a higher fluidity. This former characteristic may allow a more efficient natural flux of biological factors to the oocyte, influencing its maturation, ovulation, and safe transport to the oviduct. While the “coagulation and complement” cascades were confirmed as the prime signatures of the FF proteome, the balance between prothrombin, plasminogen, and coagulation factor XII proteins seemed crucial for the fluidity of the FF at the peak moment of the ovulatory season.

## Additional file

Additional file 1: **Table S1.** All seasonal proteome datasets, with full protein annotations. (ODS 1926 kb)
